# Retention and Separation Changes of Ternary and Quaternary Alkaloids from *Chelidonium majus* L. by TLC Under the Influence of External Magnetic Field

**DOI:** 10.1007/s10337-017-3293-3

**Published:** 2017-03-27

**Authors:** Irena Malinowska, Marek Studziński, Henryk Malinowski, Maria Gadzikowska

**Affiliations:** 1 Department of Planar Chromatography, Chair of Physical Chemistry, Faculty of Chemistry, Maria Curie-Skłodowska University, Maria Curie-Skłodowska Sq. 3, 20-031 Lublin, Poland; 2 Vexler and Baldin Laboratory of High Energy Physics, Joint Institute for Nuclear Research, Dubna, Russia; 3 Department of Inorganic Chemistry, Chair of Chemistry, Pharmaceutical Faculty with Medical Analysis Division, Medical University of Lublin, Lublin, Poland

**Keywords:** Magnetic field, Magnetochromatography, TLC, Plant extract, Alkaloids

## Abstract

Thin layer techniques proved their usefulness in the analysis of plant extract material. Thanks to their low operation costs, high sample throughput and possibility to gather chromatographic data for the whole sample in a single act, they allowed to find, and in some cases identify, active ingredients often hidden in sophisticated plant extract matrices. It was also proved that the presence of a magnetic field changes the retention of some substances, and moreover changes the separation efficiency of chromatographic systems. In the presented experiment, retention, efficiency and separation abilities of TLC chromatographic systems for mixtures of alkaloids under the influence of magnetic field depending on inductivity of the magnetic field, the saturation of chromatographic chamber and quantity of chromatographed substances were investigated. The results obtained were tested on real plant extracts that revealed the ability of chromatography in a magnetic field for separation of ternary and quaternary alkaloids from *Chelidonium majus* L. Our experiments proved that the presence of magnetic field induction lines perpendicular to the chromatographic plate plane can influence the width and retention of chromatographed substances, and can be considered as a tool for separation adjustment of plant extracts containing ternary and quaternary alkaloids.

## Introduction

Magnetism is one of the basic forces driving the Universe. Its presence influences many processes in micro- and macro-scale. The chemical compounds differ from each other in their magnetic properties. The natural consequence of this fact is the application of magnetic fields for separation of the various mixtures. The first practical application of magnetic field for separation dates back to 1792, when Wiliam Fullarton employed the field for separation of iron minerals [1]. A significant growth of interest in using the magnetic field as a separation factor falls in the 70s of the twentieth century when HGMS (high-gradient field magnetic separation) was invented and introduced for material purification, and pollution treatment [2–5]. In the following years, magnetic field was applied in many other separation applications in disciplines such as medicine, biology and biotechnology [6–8].

Besides of the above, it was also discovered that the field presence can also alter the interface interactions and processes taking part in living organisms [9]. An investigation on that phenomena can be carried out with the use of chromatographic methods. Thin layer chromatography seems to be a good choice for the research on this subject because of the modification of the planar chromatography system to work in magnetic field is relatively easy. In other point of view, thin layer techniques proved their usefulness for analytical and fundamental investigations due to their low operation costs, high sample throughput and possibility to gather chromatographic data for the whole sample in a single act. They also allow to find (and in some cases identify) active ingredients hidden in complex plant extract matrices. Moreover, the sample preparation procedure is faster and not so demanding as in case of column methods.

Chromatography carried out in a magnetic field is called magnetochromatography. However, that name quite often appears in literature in relation to magnetic separation systems (e.g. [10, 11]). Real magnetochromatographic separation system for the first time was described by Barrado and co-workers [12–14]. They carried out column liquid chromatography in magnetic field using stationary phase with the surface typical for LC chromatography with magnetic core (core–shell stationary phase). In their work, investigated solutes had been derivatized with paramagnetic constituents before chromatogram development.

According to Barrado et al., the magnetic component of the force acting on chromatographed molecule is described by equation:

1 $$ F_{\text{m}} = \frac{V\Delta \chi }{{2\mu_{0} }}\nabla B^{2} , $$

where *V* is the volume occupied by the molecule, *μ*_0_ is the magnetic permeability of a vacuum, Δ*χ* is the relative magnetic susceptibility of the compound, $\nabla$ is the gradient operator, and *B* is the inductivity of applied external field in Teslas.

As it was proved before, the magnetic permeability of the solution can be calculated as the sum of magnetic permeabilities of its components according to magnetic permeability additivity law [15, 16], but the final results are influenced by molecule association, hydrogen bond formation ratio and other phenomena taking place in the liquid phase.

Besides the above, our earlier yet unpublished research showed that there is a relation between external magnetic field presence and values of components of free interphase energy, thus the field presence also changes the phenomena taking place on the liquid–solid interface. Interfacial phenomena play a great role in nature and in functioning of different organisms, thus the investigation on influence of magnetic field on interphase phenomena are, at present times, fully justified, and the chromatography seems to be a good method of investigation in this area.

In the present paper, unmodified stationary phase, and the test solutes without pre-chromatographic derivatization were used, in order to demonstrate the influence of magnetic field on the behaviour of tested solutes in regular chromatographic system.

Taking under consideration the fact that earlier investigations proved that the presence of magnetic field changes the retention of some substances [12–14, 17–19] and moreover, changes the separation ability of chromatographic systems, mentioned above experiments let us assume that the chromatographic separation performed in magnetic field using standard chromatographic system should lead to different results compared to those performed outside the field. Based on previously optimized TLC chromatographic systems [20–22], retention, spot width (*w*), and separation (*R*_s_) changes under the influence of magnetic field of ternary and quaternary alkaloids present in *Chelidonium majus* L. plant extract samples were investigated.

## Materials and Methods

The substances investigated in the experiment were identified in waste liquors after chelidonine separation from *Chelidonium majus* L. extract obtained from Wrocławskie Zakłady Zielarskie “Herbapol”. Standard solutions (*c* = 1 g L^−1^ in methanol) of alkaloids identified in those *C. majus* L. extracts were prepared basing on substances delivered by Sigma (St. Lewis, USA).

The structures and names of investigated alkaloids identified in earlier research [20–22] are demonstrated in Tables 1 and 2.

**Table 1:**
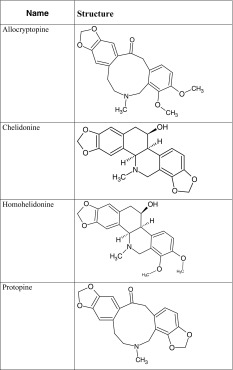
Investigated substances—ternary alkaloids

**Table 2:**
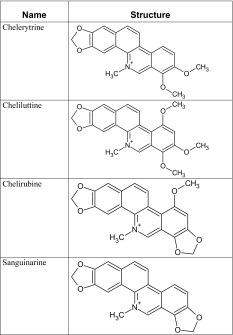
Investigated substances—quaternary alkaloids

Mobile phase composition [toluene/ethyl acetate/methanol 70/15/15 (v/v/v)] and other chromatographic system parameters (stationary phase SiO_2_ 60 TLC, distance of development 85 mm) were taken from previously published papers [20–22].

All solutes were applied on TLC SiO_2_ 60 F254 plates delivered by Merck (Darmstad, Germany) using Camag (Muttenz, Switzeland) Linomat 5 applicator as 3-mm-wide bands 5 mm from the plate edge. Chromatograms were developed in the magnetic field and outside it simultaneously in exactly identical conditions.

Chromatogram developments in magnetic field were carried out using TLC DS-II chamber placed in the gap between a pair of neodymium bar magnets. For that purpose, all metal parts from the stock DS-II chromatographic chamber were removed. Chromatographic chamber and plate saturation lasted 30 min, and was performed by placing 5 mL of mobile phase inside the internal chamber area. The saturation procedure was always performed outside the external magnetic field. The magnetic field was induced using a pair of neodymium bar magnets produced by ENES (Warszawa, Poland).

The permanent magnets used in the experiment are described by following parameters:

- dimensions: 20 mm × 50 mm × 100 mm,
- bHc coercion—min. 8992 A m^−1^ (11.3 kÖe),
- jHc coercion—min. 955 A m^−1^ (12 kÖe),
- energy density—286–303 kJ m^−3^,
- max. working temperature 353.15 K.

The outside field chromatograms were developed using standard DS-II (Chromdes, Lublin Poland) horizontal chamber made of PTFE.

After chromatogram development plates were derivatized using Dragendorff reagent. The reagent was sprayed onto the plate by Merck (Darmstadt, Germany) TLC Sprayer. Plate images were acquired using Baumer-Optronics DXA252 camera with Camag Reprostar 3 and WinCats ver 1.4.3 software by Camag. Digital images of plates were evaluated using Camag VideoScan v. 1.09 software (Camag).

Chromatograms were obtained using CAMAG TLC Scanner 3 densitometer.

In our experiments 0.25 and 0.44 T inductivities were tested. Further enhancement of magnetic field inductivity was impossible due to the TLC chamber thickness. Exact technical information considering the magnetic field generating device is available elsewhere [17, 18]. All experiments were repeated at least three times, all blunders were rejected. All chromatograms were developed in room temperature of 21 °C (294 K).

## Results and Discussion

As was mentioned before [17–19], the presence of magnetic field influences interaction between chromatographed solutes and mobile phase, the solute and stationary phase and between stationary and mobile phase. In consequence, differences appear between retention and peak shape in the magnetic field and outside the field of chromatographed extract ingredients.

In the first part of experiment, standard solutions composed of ternary and quaternary alkaloids earlier identified in *C. majus* L. plant extracts were used [20–22]. The influence of the magnetic field on retention of earlier described solutes was investigated using two analogical chromatographic systems in saturated and unsaturated chambers. The changes in chromatographic parameter in the field with lower induction (0.25 T) were smaller and more irregular than standard deviation of results obtained outside the generated field, thus it was assumed that there is no effect of magnetic field presence in the experiment.

In order to better describe differences between results obtained in magnetic field and outside it ∆*R*_M_ value was introduced. It can be calculated from Eq. (2) which is formulated as follows:

2 $$ \Delta R_{\text{M}} = R_{\text{Mmag}} - R_{\text{Mnmag}} , $$

where *R*_Mmag_—*R*_M_ value obtained for considered extract ingredient obtained during the development carried out in magnetic field and *R*_Mnmag_—*R*_M_ value obtained for considered extract ingredient obtained during the development carried out without presence of external magnetic field.

**Fig. 1:**
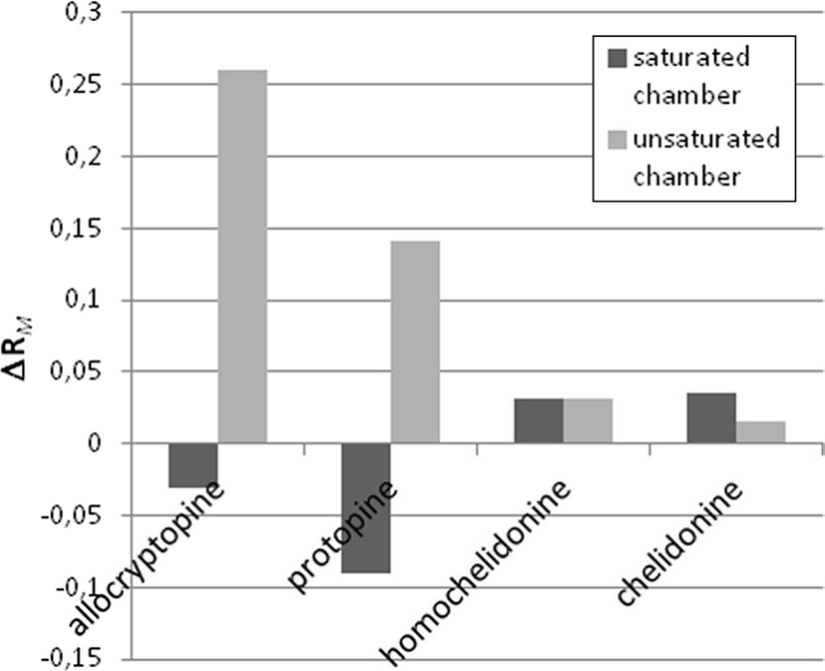
Comparison of differences between *R*_M_ values (Δ*R*_M_) obtained for chromatogram development carried out in magnetic field and outside it for saturated and unsaturated chamber. Separated compounds masses—1 μg

Changes of *R*_M_ of investigated ternary alkaloids (Δ*R*_M_) in magnetic field in unsaturated chromatographic chamber depend mainly on the evaporative component of mobile phase flow, which in the generated field are different than outside it. For all chromatographic ternary alkaloids in unsaturated chambers ∆*R*_M_ values are positive, leading to the conclusion that retention of the solutes in magnetic field is stronger than outside it. The phenomenon can be explained by more intensive evaporation of more volatile (in our case also with higher elution strengths—methanol and/or ethyl acetate) of the mobile phase components, so retention of the solutes increases (Fig. 1), which was reported in our earlier work [18].

It is unlikely that in the saturated chamber any relations between retention and ∆*R*_M_ values can be observed. For the stronger retained pair of extract ingredients (allocryptopine and protopine) negative values of ∆*R*_MS_ were observed, for homochelidonine and chelidonine negative values of that parameter were obtained. Positive values of ∆*R*_MS_ calculated for higher migrating pair of alkaloids can be interpreted that they are “attracted” stronger by mobile phase than stationary phase in magnetic field than outside it. Thus, it may be assumed that in this separation, interactions between chromatographed solute and stationary phase have greater contribution than those between chromatographed solute and mobile phase.

**Fig. 2:**
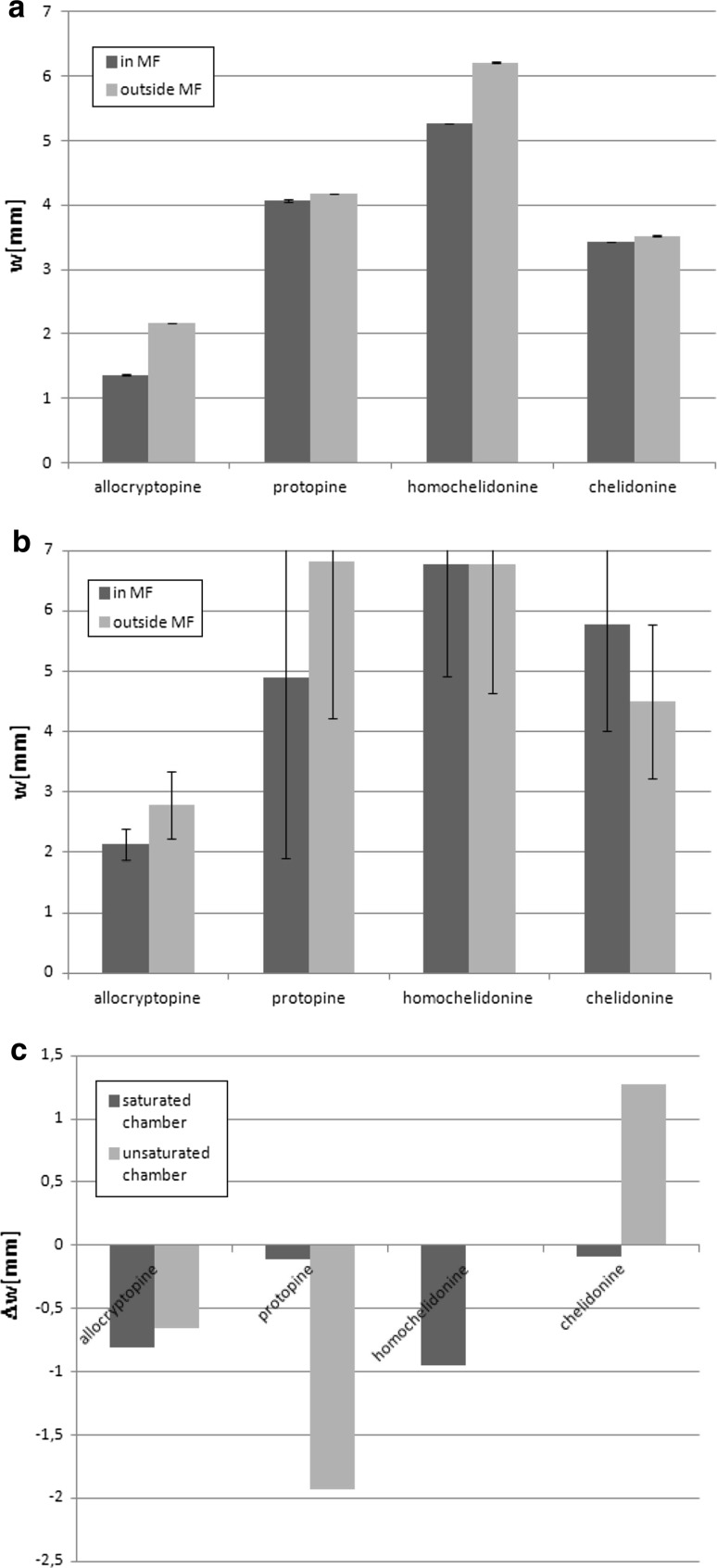
Spot widths in millimetres obtained after performing saturated (**a**) and unsaturated (**b**) chamber chromatogram development of ternary alkaloid fraction from *Chelidonium majus* L. extract with error bars, after Dragendorff reaction ‘in MF’—results obtained in magnetic field ‘outside MF’—results obtained outside magnetic field. (**c**) Comparison of differences between spot widths obtained for chromatogram development carried out in magnetic field and outside the field for saturated and unsaturated chamber. Separated compounds masses—1 μg

The next investigated aspect was the influence of magnetic field on spot width. In our previous experiments, changes in this parameter under the influence of magnetic field were observed. The differences between spot widths obtained during chromatogram development in magnetic field and outside it in saturated and unsaturated chromatographic chamber are depicted in Fig. 2a–c. ∆*w* parameter was calculated in the same way as ∆*R*_M_ parameter—by subtracting spot width (in millimeter) obtained outside the field from the spot width measured after chromatogram development in the field.

In case of saturated chamber, observed changes are larger than standard deviations and mean spot widths are lower in magnetic field for all investigated ternary alkaloids, leading to the conclusion that in case of this particular chromatographic system, magnetic field reduces the diffusion of chromatographed substances along the sorbent bed, but at present the authors are not able to interpret the reason of that phenomenon.

In the experiment performed using unsaturated chamber, decrease of mean spot width can also be observed for the considered alkaloids except the chelidonine, but the data obtained are not so obvious as in case of saturated chamber experiment, due to high standard deviations. It can be explained by unequilibrated chromatographic conditions—a standard phenomenon in thin layer chromatography. The decrease of spot width is extremely high especially for protopine, which is connected with (Fig. 1) quite intense retention decrease. It leads to the conclusion that the change of mobile phase composition caused by its evaporation to internal chromatographic chamber space strongly influences its interactions with stationary and mobile phase. It can be also suspected that protopine due to the effect of magnetic field was placed on one of demixion fronts.

Retention and spot width changes of extract ingredients influence separation efficiency of the investigated chromatographic system. *R*_s_ values calculated for chromatogram developments carried out in saturated and unsaturated chamber are presented in Table 3 Positive ∆*R*_s_ values proved that applying external magnetic field for both experiments performed using saturated and unsaturated chamber resulted in the improvement of resolution. Higher relative magnetic susceptibility of protopine, and in consequence better separation from allocryptopine did not result in worsening its separation from homohelidonine, neither in case of experiment carried out in unsaturated nor in saturated chamber (both peaks were already well separated (*R*_s_ ≫ 1.5). Similar phenomenon can be observed in case of unsaturated chamber for allocryptopine/protopine pair. Nevertheless, in case of saturated chamber, applying an external magnetic field resulted in raising separation coefficient from about 0.7 to more than one, which in connection with better repeatability of *R*_F_ and spot widths in case of saturated chamber made it a useful tool for separation of investigated compounds.

**Table 3:** *R*_S_ values calculated for neighbouring ternary alkaloids after Dragendorff reaction for chromatogram developments in saturated and unsaturated TLC chamber, respectively

	Unsaturated chamber		Saturated chamber	
	In MF	Outside MF	In MF	Outside MF
all/prot	2.40	2.23	1.06	0.70
prot/hom	3.72	2.95	8.48	8.05
hom/cheli	1.10	1.10	1.34	1.22

Separated compounds masses—1 μg

**Fig. 3:**
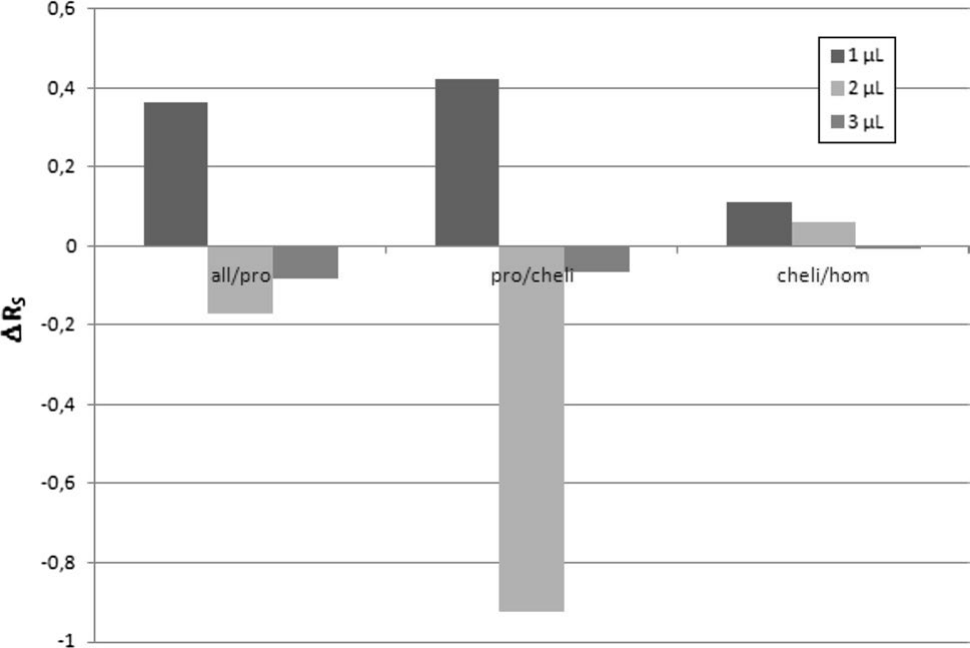
Differences between *R*_s_ values calculated for neighbouring ternary alkaloids peaks for 1, 2, 3 μL spots of chromatographed solutes in saturated chamber

Next experiment concerned the influence of the change of separated compounds quantities on the effect of magnetic field on separation ability. Table 4 shows *R*_s_ values calculated for different amounts of standards solutions obtained during chromatogram development in magnetic field and outside it in saturated chromatographic chamber. In Fig. 3, the changes of *R*_s_ (∆*R*_s_) for different amounts of investigated ternary alkaloids are depicted.

**Table 4:** *R*_S_ values calculated for neighbouring ternary alkaloids peaks for 1, 2, 3 μL of investigated ternary alkaloids after Dragendorff reaction for chromatogram developments in saturated TLC chamber

Sample volume (μL)	In MF			Outside MF		
	1	2	3	1	2	3
all/pro	1.06	0.76	0.68	0.70	0.93	0.76
pro/cheli	8.48	5.92	4.94	8.05	6.84	5.01
cheli/hom	1.34	1.01	0.89	1.22	0.95	0.89

Generally, the positive effect is present in case of 1 μL of extract solution only (exception—protopine/chelidonine separation at solute volumes 2 μL). A negative effect of magnetic field presence appears for allocryptopine/protopine pair and protopine/chelidonine pair of peaks. In case of chelidonine/homochelidonine pair the decrease of *R*_s_ gain caused by presence of magnetic field can be observed, which can be interpreted as another proof for the thesis that the presence of magnetic field modifies interaction between stationary phase and chromatographed substance (low retention = negative *R*_M_ = weak interaction with stationary phase). It may be expected that the cause of that phenomenon lies in the magnetic permeability additivity law—a high concentration of strongly diamagnetic compound makes permeation of magnetic field in the spot area impossible; however, protpine/chelidonine separation for 2 μL denies that hypothesis. That is why further investigation of magnetic field on sorption/desorption phenomena must be performed.

**Fig. 4:**
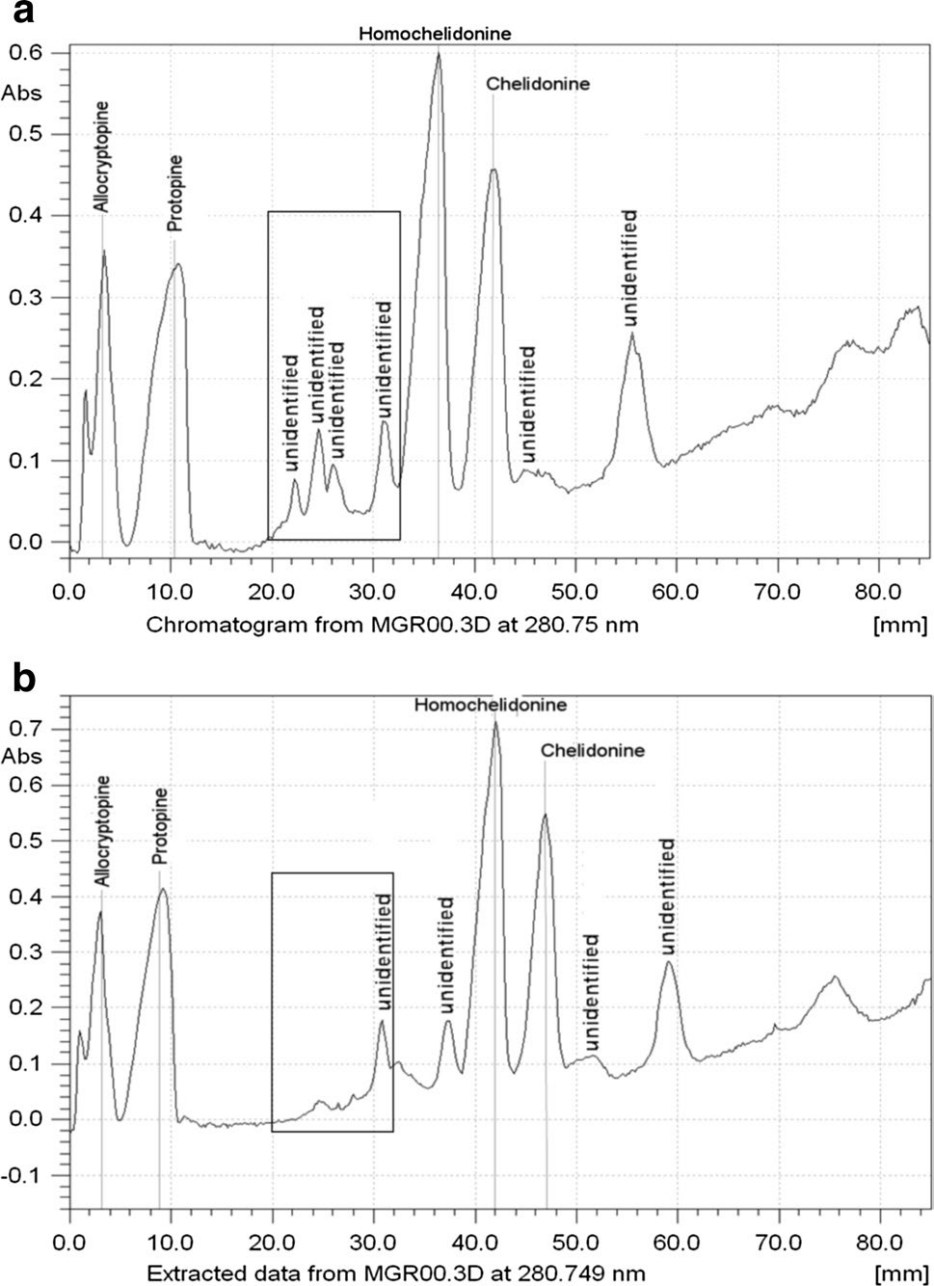
Densitograms obtained for fraction of ternary alkaloids from *Chelidonium majus* L. extract in magnetic field (**a**) outside magnetic field (**b**). 30 μL of 0.01% methanol solution applicated as 40-mm-wide band. Unsaturated chamber

Finally, the magnetic field was tested on real plant extract with identified ternary alkaloids peaks. The obtained profiles are depicted in Fig. 4a, b.

There are also some other differences between chromatograms of the mentioned plant extracts obtained in analogical chromatographic systems caused by the presence of external, perpendicular to plane plate and direction of chromatogram development magnetic field and outside it. One of them is different migration distance of the identified solutes in the extracts. Second one is different chromatogram of unidentified alkaloids on the distance between 20 and 30 mm in chromatogram obtained in magnetic field and in distance between 30 and 40 mm outside the field (marked area in Fig. 4a, b). More peaks can be observed in the mentioned range on the chromatogram developed in magnetic field compared to the one obtained outside it.

**Fig. 5:**
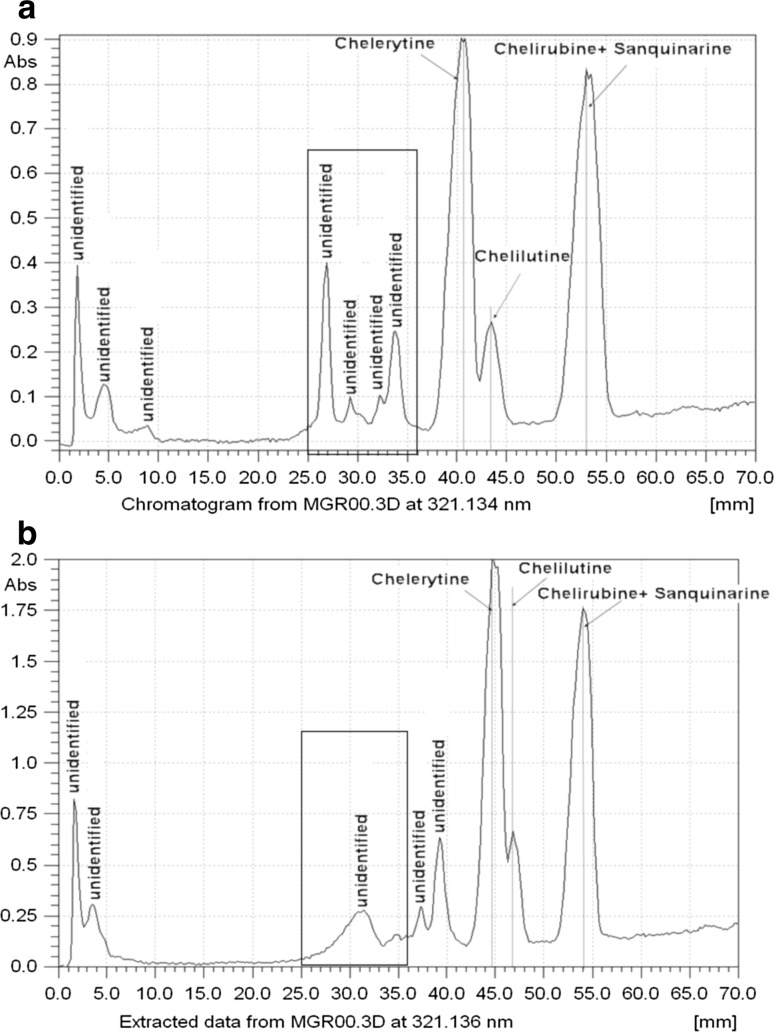
Densitograms obtained for fraction of quaternary alkaloids from *Chelidonium majus* L. extract in magnetic field (**a**) and outside magnetic field (**b**). 30 μL of 0.001% methanol solution applicated as 40-mm-wide band. Unsaturated chamber

Quaternary alkaloid fraction from *C. majus* L. extract was separated in exactly the same chromatographic conditions in magnetic field and without it as ternary alkaloid fraction; the chromatograms are presented in Fig. 4. Similar phenomena regarding changes of retention, spot width, and *R*_s_ values were observed in this experiment. The chromatograms of this alkaloid fraction are presented in Fig. 5a, b.

The appearance of additional peaks in the chromatograms obtained in magnetic field compared to the ones obtained outside it can be observed. A better separation of identified quaternary alkaloid peaks can be also observed in the migration range between 25 and 40 mm, which also proves positive influence of magnetic field on the separation of quaternary alkaloids.

## Summary

The presence of uniform, perpendicular to plane plate magnetic field influences the retention and efficiency of chromatographic system used for separation of ternary and quaternary alkaloids fraction from *C. majus* L. extract. In case of chromatogram developments carried out in unsaturated chamber, the most important change caused by the presence of the field is the change of evaporation rate of mobile phase ingredients from chromatographic plate to internal chamber space, which modifies the elution strength in a different way from a similar development carried out without the field presence. In case of saturated chamber, the most probable effect of magnetic field presence is the change of interactions between chromatographed solute and stationary phase. Moreover, the effect of magnetic field presence on retention and efficiency of separation depends from quantity of chromatographed substance. It may be considered as the proof for the thesis that the presence of magnetic field modifies the strength of surface interactions inside the chromatographic systems which causes changes of retention and width of chromatographed spots.

The phenomenon mentioned above may be used, among the others, as another cheap and easy tool for tuning the separation procedures of various mixtures of natural origin.

There are too many variables to judge the efficiency of investigated chromatographic systems. In some cases improvement and in other cases the worsening of separation have been observed. For this particular separation, applying a magnetic field regardless of the saturation of chamber improves the separation coefficient for most of the investigated neighbouring pairs of peaks, making it a useful tool for alkaloid separations and opens a way to investigate the separation of other plant extracts.

## Abbreviations

∆*R*_M_: Change of retention factor induced by external magnetic field presence
∆*χ*: Relative magnetic susceptibility
∆*w*: Spot width change induced by external magnetic field presence
∆*R*_S_: Resolution change induced by external magnetic field presence
°C: Celsius degree, temperature unit
*B*: Magnetic field inductivity
*F*_m_: Magnetodynamic force acting on molecule
HGMS: High-gradient field magnetic separation
K: Kelvin, temperature unit
Öe: Öersted, a magnetic field intensity unit
PTFE: Polytetrafluoroethylene
*R*_M_: Retention factor in TLC technique
*R*_Mmag_: *R*_F_ value obtained for considered extract ingredient obtained during the development carried out in magnetic field
*R*_Mnmag_: *R*_F_ value obtained for considered extract ingredient obtained during the development carried out without presence of external magnetic field
*R*_S_: Resolution in TLC technique
T: Tesla, a magnetic field inductivity unit
*V*: Volume of molecule
*w*: Spot width in TLC technique
*μ*_0_: Bohr magneton
